# Supplementary material for: Beta-Hydroxybutyrate (BHB), Glucose, Insulin, Octanoate (C8), and Decanoate (C10) Responses to a Medium-Chain Triglyceride (MCT) Oil with and without Glucose: A Single-Center Study in Healthy Adults
Source: Nutrients. 2023 Feb 24;15(5):1148. doi: 10.3390/nu15051148 (PMC10005646; doi:10.3390/nu15051148)
Supplement: Supplementary file 1 [file nutrients-15-01148-s001.zip › Figure S1 - Base peak intensity chromatogram of medium-chain fatty acids.pdf]

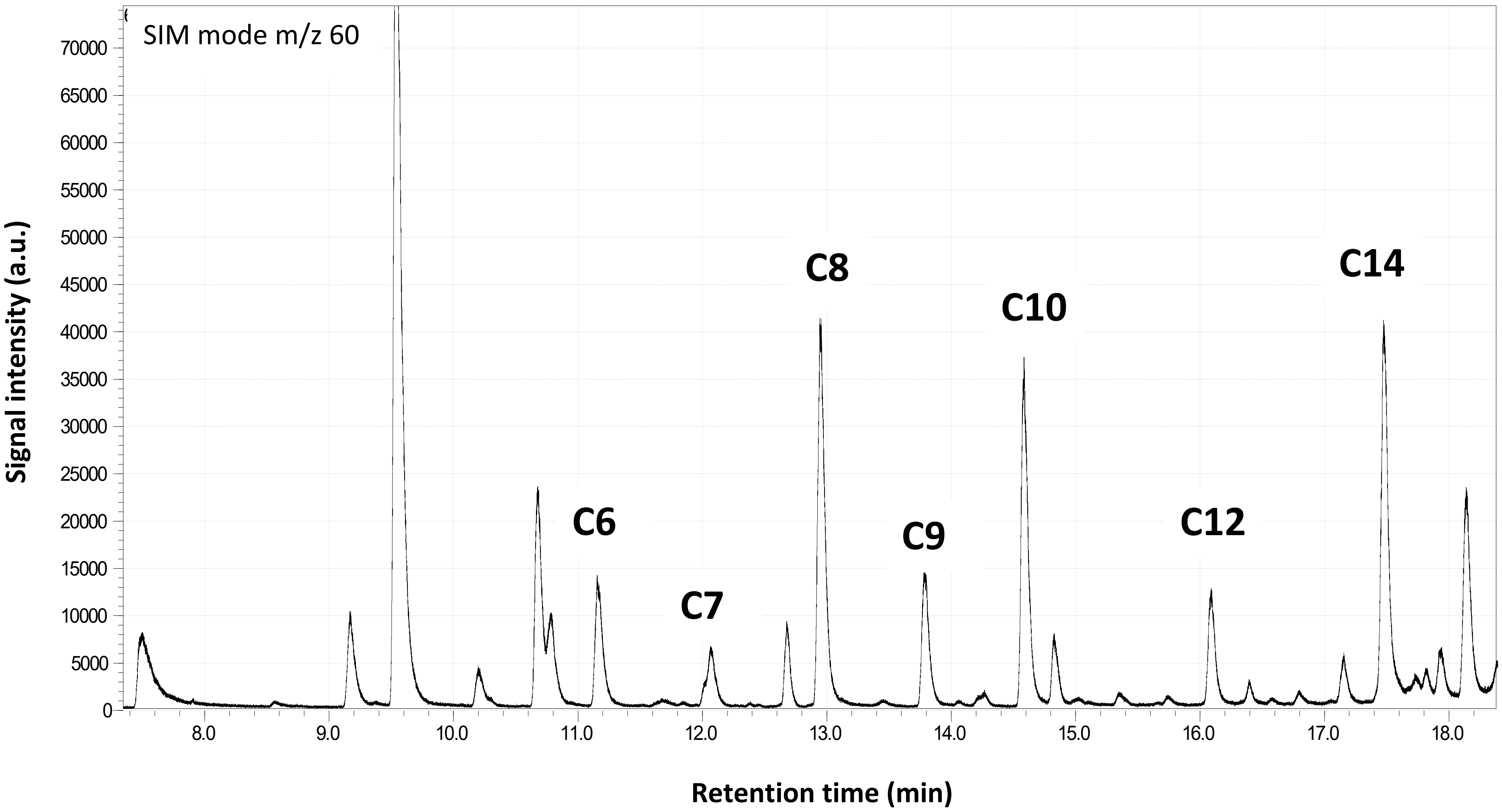

Figure S1: Base peak intensity chromatogram of medium-chain fatty acids and their labelled isotopes by gas chromatography-single quadrupole mass spectrometry.
